# Interventions to address mealtime support needs in dementia: A scoping review

**DOI:** 10.1371/journal.pone.0300987

**Published:** 2024-03-25

**Authors:** Lígia Passos, João Tavares, Melissa Batchelor, Daniela Figueiredo

**Affiliations:** 1 Center for Health Technology and Services Research (CINTESIS@RISE), Department of Education and Psychology, University of Aveiro, Aveiro, Portugal; 2 Institute of Biomedical Sciences Abel Salazar, University of Porto, Porto, Portugal; 3 Center for Health Technology and Services Research (CINTESIS@RISE), School of Health Sciences, University of Aveiro, Aveiro, Portugal; 4 School of Nursing, George Washington University, Washington, DC, United States of America; Utah State University, UNITED STATES

## Abstract

The decrease in cognitive and physical ability among people with dementia can significantly affect eating performance, resulting in mealtime support needs that could lead to inadequate oral intake, weight loss, malnutrition, and reduced functionality in activities of daily living. This scoping review aimed to identify and summarize available research literature on mealtime interventions for people with dementia, and their impact on older people with dementia living in a residential care setting, care staff, and care context/environment. A scoping review of available research published in English, French, Portuguese, or Spanish, was conducted according to the methodology established by The Joanna Briggs Institute. The search was conducted between November 2022 and February 2023 in the following databases: MEDLINE, Web of Science, Scopus, CINAHL Complete, and SciELO. A total of 275 articles were retrieved, of which 33 studies were selected according to inclusion criteria. The interventions were classified into four general categories: environmental, mealtime assistance, staff training, and multicomponent. Most studies demonstrated effectiveness in increasing oral intake and improving behaviors such as agitation and aggression in people with dementia. The impact of interventions on care staff was linked to greater knowledge and attitudes towards mealtime support needs. There is a lack of reporting on the impact of interventions on the care context/environment. Most interventions examined the effects exclusively on residents, focusing on their oral intake and behavioral patterns, particularly agitation among individuals with dementia. However, it is crucial to conduct studies that evaluate the impact on administrators, to comprehend the viewpoints of various hierarchical levels within an organization regarding challenges associated with mealtime. The findings of this scoping review can support the development of new supportive programs, or strategies to improve mealtime experience with positive impact according to the reality and needs of each person or institution.

## Introduction

Over 55 million people around the world live with Alzheimer’s disease or other forms of dementia (dementia) [1]. Dementia can be defined as a syndrome of chronic and progressive nature, that causes deterioration in cognitive function affecting memory, orientation, and language (comprehension and expression). The cognitive decline is usually accompanied and sometimes preceded by changes in mood, behavior, motivation, and emotional control [1]. Dementia also impacts functional ability and independence in activities of daily living, such as the ability to use utensils. As cognition declines, people with dementia may become unable to identify and express hunger and thirst and lose the ability to eat independently, increasing dependency on others to provide mealtime support [2, 3].

In addition to cognitive decline and functional disabilities, the environment where people with dementia eat meals can impact their performance [4]. Furthermore, the assistance provided to people with dementia plays an important role in their autonomy and independence at mealtimes. Providing excessive or needless assistance during mealtime, indifference of residents’ self-feeding capabilities, and making mealtime a task to be completed [5] can lead to excess disability, reducing residents’ autonomy and mealtime enjoyment, and triggering resistance as well as aggressive behaviors [6, 7].

According to the dictionary, eating is the act of consuming food and feeding is the act of giving food to a person, an animal, or a plant. In addition, the term mealtime refers to the time in the day when you eat a meal [8]. A medical dictionary defines feeding as giving food or administering nourishment, while eating is defined as taking, chewing, and swallowing food [9]. The terms "feeding" and "eating" are commonly treated as synonyms in literature. This usage has been noted in previous studies, where "feeding difficulties" and "eating difficulties" were used interchangeably [10]. As such, when designing and implementing mealtime interventions, it is important to distinguish between strategies or actions that support eating versus feeding needs. While "eating difficulty" in dementia can be defined as an individual manifestation of psychological and behavioral symptoms, where a person with dementia is unable to properly execute the act of eating [11], "feeding difficulty" is a wider concept, as it includes the one who is aiding the person with dementia with their meal. So, it could be defined as specific challenges faced by the care staff while feeding people with dementia. Eating and feeding are slightly related, but feeding difficulties indicate the caregivers’ problems while feeding people with dementia, instead of the problems experienced by those who are being fed [10]. In order to embrace both the concepts of eating and feeding difficulties, a broader concept is presented as “mealtime difficulties”, which contemplates environmental, social, cultural, and contextual implications with nutritional intake since mealtime involves more than the physical act of eating a meal or feeding someone [12].

The concepts of mealtime and mealtime difficulties meet the principles of the Social Ecological Model, a theoretical framework introduced by Broffenbrenner as an ecological system [13] and subsequently redefined as a model aimed at fostering changes in health-related behaviors [14]. A social ecological model applied to mealtime difficulties seeks to understand factors that influence mealtime performance in a multilevel perspective: intrapersonal (characteristics of the person with dementia), interpersonal (characteristics and skills of the caregiver, and their interaction with the person with dementia), environmental (physical and cultural characteristics of where meals take place) and institutional (caring practices and institutional policies) [15].

Considering the scope of the terms mealtime/mealtime difficulties, relying on the Social Ecological Model, and to ensure the use of a strengths-based language “mealtime support needs” will be used throughout this article.

The decrease in cognitive and functional ability can significantly affect eating performance, and older people with dementia living in residential care settings (residents), especially in the late stages of the condition, can develop mealtime support needs. These individuals may be unable (partially or completely) to start or keep attention to mealtime tasks, take the food to the mouth, chew, or swallow, or have behaviors such as apathy, wandering, refusal, or indifference during mealtime [10]. The consequences of mealtime support needs can lead to a range of complications, from weight loss, malnutrition, and dehydration to caregiver stress and less opportunity to socialize [16, 17].

As dementia progresses, the ability to self-perform activities of daily living, including eating/ self-feeding, combined with the unavailability of family members to provide continuous care often leads to institutionalization [18]. The incidence of institutionalization varies throughout the world, and it is estimated that in industrialized countries about 2 to 8% of people over 65 years of age reside in nursing homes, and this number is expected to increase hereafter [19]. It is estimated that 45% of residents have dysphagia, and 40 to 86% experience some feeding challenges [20, 21]. Mealtime support needs could be related to the behavior symptoms of dementia, inappropriate food consistency, inadequate posture and positioning during meals, fast feeding supply by the caregiver, and due to cognitive, functional, and environmental conditions [22].

The quality of care provided by nursing homes staff and the mealtime environment can contribute to better results in food acceptance [4, 23–25]. Staff who are more aware of poor food intake and an enabling dining environment create a better mealtime experience and therefore improve the nutrition and hydration of residents [4]. Enhancing care staff training in mealtime support needs for people with dementia is crucial, as current programs often prioritize mechanical feeding skills over comprehensive strategies for residents’ participation and communication. A recent review showed that formal caregivers require additional training and education to effectively manage diverse challenges encountered during mealtime [26].

Mealtime support needs in people with dementia have been described in the literature since the early 1990s, and the first evidence-based studies on the effectiveness of interventions appeared more than ten years later [27]. A broad number of interventions were created to improve the nutrition of residents, and each one focuses on distinct factors that may contribute to a better food and fluid intake, as well as on controlling the behavioral and psychological symptoms of dementia [28]. A literature review reported the existing interventions as changed mealtime delivery service and staff allocation patterns, adaptation of food texture, occupational therapy and behavioral interventions, verbal cueing, and dining environmental changes [29].

Despite the number of existing interventions, further research is needed to identify the most effective interventions and inform the mealtime support strategies that should be adopted by care staff [27, 30, 31]. Previous reviews focused on identifying and evaluating existing interventions to minimize mealtime support needs, but to the best of the authors’ knowledge, there are no publications that seek to relate the impact of these interventions on the social-ecological perspective, with an integrated view of the impact on residents and care staff, and from the nursing home administrators’ perspective on the impact of these interventions. The social-ecological model embeds individuals in a broad social system and outlines their interactive characteristics with underlying environments as influencers of health outcomes [32]. The framework considers individual and environmental determinants, supporting the development of systematic intervention mechanisms capable of influencing behavior changes across various levels of influence, including applicability to mealtime support needs [19].

This scoping review aimed to identify and summarize available research literature about mealtime interventions for older people with dementia living in a residential care setting. By adopting a social-ecological perspective, this review seeks to not only summarize the outcomes for residents but also comprehensively examine the impact of these interventions on care staff and the care environment, considering the viewpoints of nursing home administrators.

## Methods

### Design

This scoping review was conducted following the methodology established by The Joanna Briggs Institute (JBI) [33] and its protocol was registered with the International Platform of Registered Systematic Review and Meta-Analysis Protocols (INPLASY) on 04 August 2021 (registration number INPLASY202180015) [34].

### Search strategy

A search strategy was developed to identify published and unpublished studies. The first reviewer (LP) searched the following electronic databases: MEDLINE (via PubMed), Web of Science, Scopus, CINAHL Complete (via EBSCO), and SciELO, using a combination of keywords and MeSH terms, along with Boolean operators. The search limits were applied for title/abstract.

The search strategy was reviewed by a university librarian and was adapted according to the search patterns of each database. The following search terms were used: (dement* OR alzheimer*) AND (“older people” OR “old* person” OR elderly OR aged OR senior)) OR (caregiv*OR “formal caregiver” OR “direct care worker*” OR staff* OR nurs*) OR (manager*OR “health manager” OR administrator OR “nurse administrator” OR “nursing home administrator” OR “ALF administrator” OR “assisted living administrator”) AND ((intervention* OR train* OR program*) AND (“feeding difficult*” OR “eating difficult*” OR “mealtime difficult*” OR “mealtime challenge*” OR “mealtime management”)) AND (“nursing home*” OR “long term care” OR “care home*” OR “residential facility” OR “home* for aged” OR “residential home” OR “elderly care” OR “residential care” OR “assisted living facility”).

A manual search for systematic review articles in the databases Cochrane, JBI, and PROSPERO was undertaken to identify additional papers of interest. The search on grey literature included DARTEurope, OpenGrey, and *Repositório Científico de Acesso Aberto de Portugal* (RCAAP).

Articles written in English, French, Portuguese, or Spanish, published from 1990 onward were considered for inclusion.

### Eligibility criteria

This scoping review included studies with interventions designed exclusively for mealtime support needs and implemented and evaluated in residential care settings. Studies needed to include as participants older people with dementia (all types or degrees/stages) living in a residential care setting (residents) who were 60 years or older, care staff, and facilities administrators. Studies with residents in tube feeding (exclusive or not) were excluded, since it could indicate issues associated, such as dysphagia or very advanced dementia (even tube feeding is not recommended at this stage [35]), or other impairments in addition to mealtime support needs. Interventions developed for hospitalized or people with dementia living in the community were excluded, as well as those with participation of informal caregivers. Studies in which interventions were merely nutritional or pharmacological were also excluded.

This scoping review considered quantitative (experimental or quasi-experimental, and observational studies), qualitative, and mixed-method studies. Study protocols, conference abstracts, letters, and correspondence reports were excluded. Studies that were not accessible via university agreements, or even be made available after contact with the authors were excluded.

### Study selection

All databases were searched and articles identified, then duplicates were removed. Two reviewers (LP and JT) screened titles and abstracts based on the inclusion criteria. Disagreements were resolved by the fourth reviewer (DF). Then, the studies were retrieved, and the full texts were read. Full-text studies that did not go through the inclusion criteria were excluded. The findings were reported using the Preferred Reporting Items for Systematic reviews and Meta-Analyses extension for Scoping Reviews (PRISMA-ScR) [36].

### Data extraction

Data were extracted by the first author into a Microsoft Excel spreadsheet and, afterward, verified by the second and fourth reviewers. The data extracted included specific details about authors and year of publication, country, study aim and design, intervention description, sample size and characteristics, primary outcomes (for residents, staff, and care environment), and main results.

### Data analysis and synthesis

As recommended by JBI [33], data extracted from the included studies are presented in both diagrammatic and tabular form, and in a descriptive format to address the review question and objective. In order to organize the description of the results, four general categories of interventions were determined, according to the strategies used by the authors: environmental, mealtime assistance, staff training, and multicomponent.

## Results

### Selection process

**Fig 1:**
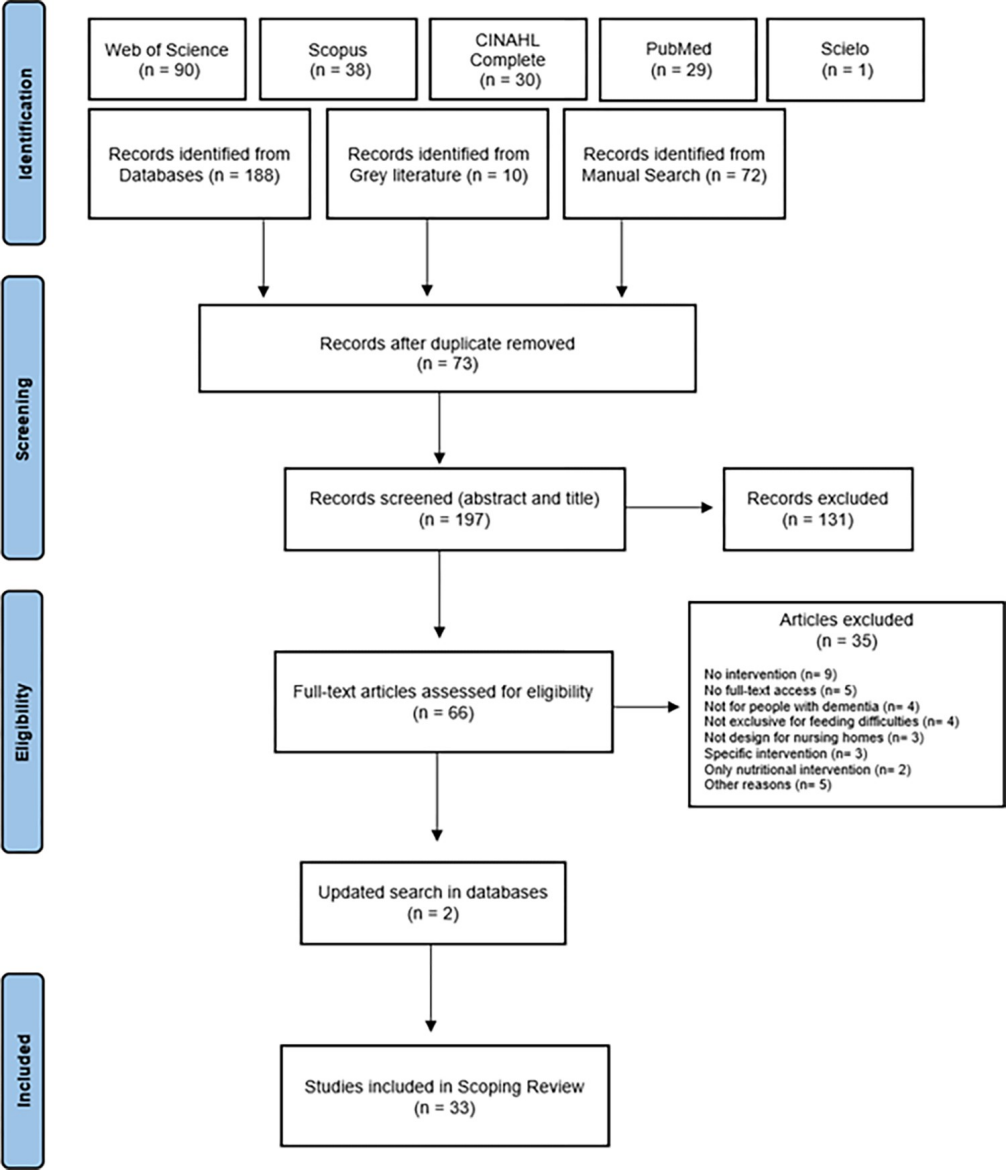
Flowchart of the selection process.

The database search was carried out between November 2022 and February 2023, and resulted in 193 potentially relevant studies and a manual search, including grey literature and articles references, resulted in 82. A total of 275 studies were reached, and after duplicates removal, 200 studies remained for additional analysis. After screening titles and abstracts, 69 full-text papers were retrieved, 33 of which were included in the final scoping review (Fig 1). Those excluded did not describe an intervention (n = 9), the full text was unavailable (n = 5), the intervention was not designed for people with dementia (n = 4) or was not exclusive to intervene on mealtime difficulties (n = 4), or not performed in nursing homes (n = 3). Three interventions were designed for specific problems, such as dysphagia (n = 2) or hyperphagia (n = 1), or were interventions focused only on nutritional supplementation (n = 2). Six studies were excluded for other reasons, like being from 1986, a conference abstract, a PhD thesis whose paper was already included, design process of an assistive robot, or interventions including informal caregivers or research assistants.

### Characteristics of included studies

The 33 included studies were published between 1995 and 2023 and conducted in nine countries: the United States (n = 13), Canada (n = 6), Taiwan (n = 6), Sweden (n = 3), Australia (n = 1), United Kingdom (n = 1), South Korea (n = 1), France (n = 1), and Japan (n = 1). In 75.7% (n = 25) studies the participants were only residents, while in 21.2% (n = 7) the participants included both residents and nursing staff. Only one study (3.1%) considered the participation of residents, nursing staff, and nursing home administrators. The analyzed studies had a total of 1062 participants, of which 880 were residents, 177 care staff, and 5 nursing home administration. Most of the participants were women, and the mean age of the residents was 80.07 years old.

### Study design and outcome measures

The most common study design was the experimental (54.5%; n = 18), such as pretest-posttest, feasibility, prospective, time-series, RCT, followed by quasi-experimental studies (24.2%; n = 8), case studies (9.1%; n = 3), mixed-methods—pre-posttest + focus group or interviews (6.1%; n = 2) and observational (6.1%; n = 2).

Most interventions had two predominant primary resident outcomes: improving residents’ meal intake and reduction in residents’ challenging behaviors during mealtime. Few studies have focused on the mealtime experience or person-centered care.

#### Oral intake measures

Measures for meal intake included caloric intake calculations, tray weights, number and amount of snacks/ supplements. Interventions based on changes and adaptations in the environment used the residents’ food intake as a measure of effectiveness, by calculating the calories ingested [37–42], carried out with specific software, or by weighing the meal before and after consumption [38, 43–47]. A visual estimate of the percentage of food ingested from each meal was also used [41], as well as the number of snacks consumed [48]. Mealtime assistance interventions estimated food intake through consumed meal weights [49, 50] and by the visual percentage of food ingested [49]. Staff training and multicomponent interventions likewise used the proportion of food ingested by weight before and after the meal [51–54], as well as the caloric intake and visual percentage of intake [55].

#### Resident challenging behavior measures

Measures for residents’ challenging behaviors included, in the group of environmental interventions, an evaluation of agitation, using The Cohen-Mansfield Agitation Inventory (CMAI) [56–59]. Among the mealtime assistance, staff training, and multicomponent interventions, the assessment of the feeding behaviors of residents predominates, either using EdFED [51–53, 55, 60] or the Eating Behavior Scale [17, 60].

### Environmental interventions

The type of intervention most frequently found among the studies analyzed was that of an environmental nature (54.5% n = 18). Changes in the environment include adaptations at the level of inclusion of stimuli, such as light and noise levels, and the improvement of the environment of a dining room, use of sound, as well as changes in meal service delivery or food presentation (Table 1).

**Table 1:** Environmental interventions studies (n = 18).

Study	Design	Aim	Population	Intervention	Outcome Measure(s)	Main results
Brush et al., 2002 (USA)	Pre- post-intervention	To examine the effect of improved lighting and table setting contrast on residents’ oral intake and behaviors during meals	25 residents (22 women and 3 men); 70+ years old Inclusion criteria: physician diagnosis of dementia, ability to independently feeding or require minimal assistance, and consistent attendance in the dining room for at least two meals a day	Facility 1: two 150-watt halogen lights + all chandeliers turned on during meals, and two additional fluorescent tubes. Navy blue tray liners were added under the plates to increase contrast at the table setting Facility 2: clip-on reflectors + fluorescent fixtures + two 150-watt halogen light fixtures. The tables were covered in dark green nonglare tablecloths to cover the peeling finish and reduce glare, and navy-blue tray liners were added to create contrast with the white plates	Caloric intake Percentage Lighting MAST COMFI	23 out of 25 residents experienced an increase in caloric intake after the lighting and contrast intervention In Facility 1, total COMFI scores increased significantly (p < .05) from 54 at baseline to 74 at posttest. MAST scores remained consistent from baseline (10.7) to posttest (10.8) In Facility 2, COMFI scores increased from 48 to 60 (p < .115), and MAST scores at Facility 2 decreased from 6.2 to 4.8 (p < .331) At Facility 2, where the lighting changes were most dramatic, the staff felt that they themselves had experienced the most positive changes
Chang et al., 2010 (Taiwan)	Quasi-experimental time series	To set up a music program during lunchtime and to assess whether there was an effect on the resident’s level of behavior problems	41 residents (26 women, 15 men) Mean age: 81.69 years Inclusion criteria: 65+, diagnosed with dementia, MMSE ≤ 23, previous display of problem behavior, no hearing impairment, not a music listener while at the Nursing Home, not bed-bound	Nature music (music from a single piano and nature sounds such as bird song, whale song and running water), during lunchtime (11-12h), 60-65dB 8-week time series: 4 weeks with music, and 4 weeks without music	Barthel ADL MMSE CMAI	Music program reduced, significantly, physical and verbal aggressive behavior among residents There were no significant changes in the overall CMAI score and the verbally non-aggressive score There was a one-week time lag between the implementation of the music program and a significant effect on the residents
Charras & Frémontier, 2010 (France)	Experimental	To study the impact of changed mealtime experiences in nutrition and food intake of people with Alzheimer-type dementia	18 residents with Alzheimer-type dementia Mean age: 85.19 years Experimental group: n = 8 Comparison group n = 10	The intervention consisted of staff sharing lunchtime meals with residents, help them, when necessary, ensure there is sufficient time, everyone is sitting comfortably and there is proper equipment available. The residents should be the focus of attention	MMSE Body weight Observations of the staff	Significant weight gain among participants of the experimental group (3.37kg) and a significant weight loss in the control group (2.22kg) Staff observations (focus group): residents became more independent to feeding themselves and regulating their food intake, more interactions between residents with other residents, and residents with staff Less burnout for staff and better understanding about residents eating behaviors
Denney, 1997 (USA)	Quasi-experimental time series	To report observations and quantify changes in the incidence of mealtime agitated behaviors in residents who were exposed to quiet music	9 residents (6 women, 3 men) Mean age: 74.8 years Inclusion criteria: physician documented diagnosis of dementia	Relax With the Classics: Volume 1, Largo and Volume 2, Adagio (1987) was played at lunchtime, every day of the music week Weeks 1 and 3: no music, Weeks 2 and 4: music	CMAI	Reduction of 46% in the incidence of agitated behaviors from baseline to the end of the first week of music. 37% of decrease in the fourth week (with music) and 31% from week 3 (no music) The behaviors most changed were verbally agitated behaviors and physically non-aggressive behaviors.
Desai et al., 2007 (Canada)	Experimental	To compare energy intakes in residents receiving meals by bulk (cafeteria style with waitress service) vs traditional tray delivery systems and determine residents’ characteristics that identify responsiveness to type of foodservice provided	26 residents (tray foodservice): Mean age 86.2 22 residents (bulk foodservice): Mean age 88.8 Inclusion criteria: diagnosis of probable Alzheimer Disease, ability to consume meals independently or require only minor assistance	For 21 consecutive days, one facility delivered tray meal service and a new other delivered a bulk meal service + environmental	Body mass index London Psychogeriatric Rating Scale Weighed food intake Meals’ nutrient profile (Dietary Food Management Software)	Higher 24-hour total (P<0.001) and dinner (P<0.001) energy intakes in residents receiving bulk compared to tray delivery were predominantly associated with greater carbohydrate intakes (P<0.001) Higher energy, carbohydrate, and protein, but not fat intakes, with bulk delivery were more apparent in individuals with lower body mass indexes
Dunne et al., 2004 (USA)	Pre and post-intervention	To examine how tableware contrast manipulations may affect food and liquid intake	9 participants (all men) Mean age 82.7 years Inclusion criteria: ability to eat independently	Baseline: white plates and cups, stainless-steel flatware Intervention: high-contrast red plates, red cups and flatware Post-intervention: plates, cups and flatware from the baseline Follow-up (1 year): like the first study, but used high-contrast blue, low-contrast red and low-contrast blue tableware	Food and liquid intake MMSE	8 of 9 participants increased food intake in 25% and liquid intake in 84% during the high contrast intervention versus baseline condition In the follow-up study, the high-contrast intervention (blue) resulted in significant increases in food and liquid intake; the low-contrast red and low-contrast blue interventions were ineffectual
Edwards & Beck, 2002 (USA)	Time series	To quantitatively examine the influence of aquarium observation on nutritional intake and changes in body weight of residents	62 residents (38 women, 24 men) Mean age: 80.1 years	Treatment: fully self-contained automated aquariums with colorful fish were introduced into the activity/dining area. Control: a scenic ocean picture was introduced	Body weight Nutritional intake	Nutritional intake increased (21.1%; p < .001) when the aquariums were introduced and continued to increase during the follow-up Weight increased (1.65 lbs; p < .001) over 16 weeks Participants required less nutritional supplementation, resulting in health care cost savings
Edwards & Beck, 2013 (USA)	Prospective observational	To assess whether residents who observe aquariums in the dining facilities increase the amount of food they consume and maintain body weight	70 residents (52 women, 18 men) Mean age: 82.2 years Inclusion criteria: diagnosis of dementia, no diagnosis of terminal/end-stage disease, ability to take nutrition by mouth	Introduction of the aquarium “The Rolling Sea” into the common dining room for 8 weeks	Body weight Food intake at each meal	A total increase of 196.9 g of daily food intake (25.0%) was noted from baseline to the end of the study Resident body weight increased an average of 2.2 pounds Eight of 70 residents experienced a weight loss
Engstrom & Hammar, 2012 (Sweden)	Experimental single-case	To describe whether caregivers’ humming during lunch time affects eating and feeding problems of residents	2 women with severe dementia, fed by staff, living at the nursing home for more than 20 weeks, MMSE score 0	Baseline (2 weeks): staff fed residents as they usually do, without humming Intervention (weeks 3 and 4): staff was instructed to hum sing-along songs, children’s songs, and popular songs from the early part of the 20th century Follow-up (week 5): staff returned to a normal lunch situation without humming	Food and liquid intake MMSE EdFED	Participant #1 kept food and liquid intake almost the same during all sessions; total EdFED score decreased from a mean score of 14 at baseline to a mean score of 8.5 during the intervention Participant #2 had the meal intake during humming intervention sessions less than half of the intake during sessions without humming; total EdFED score decrease in mean score from 12 at baseline to 8.5 during the intervention
Hicks-Moore, 2005 (Canada)	Quasi-experimental time series	To examine the relationship between relaxing music and agitation in a group of residents	30 participants (21 women, 9 men) Mean age: 82.4 years Inclusion criteria: diagnosed with irreversible dementia, Alzheimer Disease or severe cognitive impairment	Weeks 1 and 3: no music was played Weeks 2 and 4: music was played during the evening meal- Relax With the Classics: Volume 1, Largo and Volume 2, Adagio (1987)	CMAI	The incidence of agitated behaviors observed in the 4 dimensions measured decreased in the weeks that music was played
Ho et al., 2011 (Taiwan)	Single group pretest-posttest	To evaluate the effectiveness of researcher-composed music on residents’ agitation	22 participants (12 women, 10 men) Mean age: 77.27 years Inclusion criteria: no hearing impairment; resident for more than 3 months; 65+ years; MMSE equal to or lower than 23, CMAI score 35 or higher, not be bed bound, speak Mandarin or Taiwanese	Six piano pieces were played at mealtimes twice a day, 7 days a week, for 1 hour during lunch, and 1 hour for dinner, for four consecutive weeks Music volume was chosen to be 55–70 dB	CMAI Likeability of the music	The global CMAI scores had declined by 29.1% of baseline at T5. All four sub scores of CMAI had also gradually decreased by 25.09%–35.91% of baseline by T5 The four components of the CMAI were slightly increased at T6 but still significantly lower compared with baseline data (all P > .008), indicating that the 4-week music intervention had a 2-week linger effect for agitation 68.2% (n = 15) of residents expressed that they liked the music played at mealtime
Kimura et al., 2019 (Japan)	Quasi-Experimental	To explore whether the addition of a sauce affects finger-snack intake among residents	Experiment 1: 21 residents (14 women, 7 men) Mean age: 84 years HDS-R score 0 to 17 Experiment 2: 14 residents (9 women, 5 men) Mean age 82.9 years HDS-R score 0 to 24	Experiment 1: three pieces of baumkuchen with chocolate sauce and three pieces without sauce and a cup of tea were put in front of each participant, during the afternoon snack time Experiment 2: three pieces of baumkuchen with agave sauce and three pieces without sauce and a cup of tea were put in front of each participant, during the afternoon snack time	Consumption of snacks with and without sauce	Snack consumption was greater for the with-sauce options than for the without-sauce options 90.5% of the participants in Experiment 1 and 64.3% of participants in Experiment 2 ate more snacks with sauce than without sauce
McDaniel et al., 2001 (USA)	Case study	To evaluate noise and lighting conditions at mealtimes and to assess the food intake of ambulatory residents	16 residents (01 woman, 15 men) Age from 61 to 81 years Inclusion criteria: residents must be veterans, ambulatory, at least partially continent, some comprehension of spoken language, able to assist with feeding and dressing themselves	Phase I: Extended-care (EC) - 1,762 square feet with quarry tile and ceiling fans; the television is on during each meal Phase II: Alzheimer’s’ Unit (AU) - 484 square feet with low-gloss vinyl composition tile and no ceiling fans. There is no television. Relaxing music is routinely played during meals	Weight Time to consume meals Remaining food 5-day nutritional analysis (Nutritionist IV software)	Intake of calories and protein was slightly higher, with some days significantly higher, in the AU Total five-day fluid intake at breakfast was significantly higher in the AU (p ≤ .02) Total time for meals (breakfast and lunch combined) was similar in both phases Mean weight change was not statistically significant
McHugh et al., 2012 (USA)	RCT	To systematically and empirically test a long-held clinical observations and curiosities about the impact of singing in nutritional intake of residents	15 residents (12 women, 3 men) Mean age: 86.9 years Control wait-list group (CWL)—n = 7 Vocal recreative music therapy group (VMT)—n = 8	4 days per week, for 3 weeks, residents in the VMT group, seated in a semicircle facing an electronic keyboard on a stand, listening a list of songs selected (American popular song) After each session, participants were guided to the dining room where they followed their typical routine for the mid–day meal	Lunch intake (software Care-Tracker)	There were no compelling trends in food intake In the VMT group, the mean food intake during the treatment period was slightly higher (six of eight participants) In the CWL group, most participants’ intake was higher during the treatment period than during the baseline period. However, all increases, and decreases were minor
Ragneskog, Kihlgren, et al., 1996 (Sweden)	Case Study	To investigate if and how different dinner music on a nursing home affected residents and, in this case, which type of music as best to reduce behavior symptoms	5 participants (4 women, 1 man) Mean age: 80.6 years	Week 1: collect baseline data Weeks 2 and 3: soothing, soft, melodious, relaxing, and romantic music Weeks 4 and 5: popular Swedish music from the 1920s and 1930s Weeks 6 and 7: pop and rock music from the 1980s by internationally well-know artists Week 8: control period—no music was played	List of types of behaviors Time spent with dinner (stopwatch)	Four of the 5 residents spent more time with dinner during the 3 musical periods Dinner time decreases from playing of the soothing music toward the control period Staff fed residents significantly more often when soothing music was played
Ragneskog, Bråne, et al., 1996 (Sweden)	Quasi-experimental time series	To investigate whether dinner music influences food intake and symptoms common in dementia (depressed mood, irritability, and restlessness), as well as to determine whether a particular type of music was preferable	20 participants (10 women, 10 men) Mean age: 80 years	Week 1: no music Weeks 2 and 3: soothing music Weeks 4 and 5: Swedish tunes from the 1920s and 1930s Weeks 6 and 7: control period without music	GBS scale Food intake (weight) Pulse Body Weight	During music periods residents ate more in total, especially the dessert Staff thought to be influenced by the music, as they served the residents more food whenever music was played Residents were less irritable, anxious and depressed during the music periods
Shatenstein & Ferland, 2000 (Canada)	Pre-post test	To evaluate the nutritional and clinical consequences of changing from a centralized food delivery system to decentralized bulk food portioning	22 participants (21 women, 1 man) Mean age: 81.6 years	Introduction of a decentralized bulk food distribution system during 10 weeks—each meal was portioned on resident’s floor	Anthropometric indicators (height, weight, BMI, mid-upper-arm circumference, triceps skinfold thickness, mid-upper-arm muscle circumference) Nutritional status indicators in elderly (albumin, lymphocytes, glucose, sodium, potassium, transferrin, hemoglobin and plasma vitamin B12 and plasma folate) Estimate waste food	50% of the residents gained body weight, 36% lost and 14% remained relatively unchanged All parameters except albumin and sodium levels were unchanged after intervention The average proportions consumed of food served during the 2 observation periods showed that the increased nutrient intakes during the post introduction period resulted from residents’ consumption of higher percentages of the standard portions served to them
Thomas & Smith, 2009 USA	Time-series crossover	To examine whether music played during meals, by reducing agitation, would result in increased caloric consumption among residents with middle dementia	12 residents (11 women, 1 man) Mean age: 83.5 years Inclusion criteria: diagnosis of Alzheimer Disease, adequate auditory skills, able to self-feed, at risk of malnutrition	A music selection was played at the beginning of the dining period at 12:00 to 1:30 p.m., with volume set at approximately 60 decibels. Total of 8 weeks (with alternating weeks of no music and music)	Estimation of food intake Total caloric intake (Food Processor PLUS program) Music assessment form	Overall, residents consumed 20% more calories when familiar background music was played compared to an eating environment without music During music days, residents voluntarily remained in the dining room for an extended period, and were more socially engaged compared to the no-music days

Abbreviations: MAST = Meal Assistance Screening Tool; COMFI = Communication Outcome Measure of Functional Independence; MMSE = Mini-Mental State Examination; ADL = Activity of Daily Living; CMAI = Cohen-Mansfield Agitation Inventory; EdFED = Edinburgh Feeding Evaluation in Dementia; HDS-R = Revised Hasegawa’s Dementia Scale; RCT = Randomized controlled trial; DSM-III = Diagnostic and Statistical Manual of Mental Disorders; NINCDS-ARDRA = National Institute of Neurological and Communicative Diseases and Stroke/Alzheimer’s Disease and Related Disorders Association; BMI = body mass index

Nine studies used music as a factor of change in the environment during mealtime. Three studies [57–59] evaluated the effect of classical music on residents’ agitation during meals. One study [56] associated music from a single piano with sounds of nature, such as the sound of birds, whales and rain, and observed the effect on the residents’ level of behavior problems. Another study [46] observed how staff humming influences residents’ food intake and eating/feeding abilities. The other 4 studies [40, 41, 47, 61] comprised the use of local popular songs, or a music selection based on residents’ preference, and thus assessed the impact of music intervention on food intake and behaviors (agitation, mood, irritability, and restlessness).

Changes in meal service delivery style were presented in three studies. Two studies [38, 42] evaluated the oral intake and nutritional status of residents by changing the way meals are served, testing the bulk style instead of the traditional way of serving meals on a tray. The other study [62] evaluated whether sharing mealtimes with staff impacted the resident’s food intake.

Two studies [44, 45] placed an aquarium in the dining room and looked at the effect that observing it would have on resident’s food intake and body weight. Two studies [37, 39] adjusted lighting and noise conditions in the dining room and then looked at how the changes interfered with residents’ food intake and behavior. Finally, two studies evaluated changes in oral intake by manipulating the color contrast of tableware [43] or adding a sauce to snacks consumed by residents [48].

#### Impact of environmental interventions

All intervention based on the environment’s adaptations focused their objectives only on residents, who were the only participants evaluated. The main objectives of these studies were related to the improvement of nutritional status with increased food and fluid intake, and also the control of challenging behaviors during mealtimes, such as agitation or aggression. Two studies evaluated the impact of the intervention on both dimensions.

From the 11 studies that aimed to increase the food intake of residents, 10 had positive results, demonstrating the impact of the intervention in a better and greater food intake, and in some cases even gain (or maintenance) of body weight [37–39, 41–45, 48, 62]. The other study [40] showed that oral intake was only slightly higher compared to the control group. All five studies [47, 56–59] that aimed to reduce behaviors such as agitation or aggression during mealtime demonstrated the satisfactory impact of interventions on residents, evidencing the reduction of agitation and aggression (physical and verbal). Two studies demonstrated the impact of interventions on both oral intake and residents’ challenging behavior. One study showed that the oral intake of the residents was less than or equal to the intake before the intervention while eating behaviors improved during the intervention [46]. The impact of other intervention was more positive, with residents having higher oral intake and being less irritable, anxious, and depressed during the musical intervention [47].

Although the focus of the studies is on the residents’ food intake, two studies reported the impact on care staff and the administrators’ perspective on this impact. A study presented evidence that staff showed less burnout and had a better understanding of resident’s mealtime behaviors due to the nutritional improvement and better interactions between residents and staff [62]. The findings of the other study indicated an institutional interest since residents increased their food intake and needed fewer nutritional supplements, thus resulting in health-care cost savings [44].

### Mealtime assistance interventions

Four studies (12.1%) addressed residents’ mealtime support needs through interventions focused on mealtime assistance (Table 2). In this category, interventions focused on improving staff attitudes and behaviors when assisting residents during meals. Three of them are based on staff’s behavioral attitudes when helping residents with meals [50, 63, 64]. One study tested the effects of the use of a nursing intervention on mealtime behavior of people with dementia who wander assessing the frequency of table leaving as well as food intake and body weight [49]. Using a social reminiscence protocol or structured verbal cueing protocol, another study assessed the effects of reminiscence therapy on the residents’ consumption of food [50]. With behavioral strategies such as directed verbal prompts and positive reinforcement, another study evaluated changes in the level of eating independence [63]. Also, to promote functional feeding, using two interventions (one contextual and one behavioral) a study evaluated its impact on resident’s nutritional status [64].

**Table 2:** Mealtime assistance interventions studies (n = 4).

Study	Design	Aim(s)	Population	Intervention	Outcome Measures	Main results
Beattie et al., 2004 (USA)	Multiple case study	To determine the effect of the systematic use of a behavioral nursing intervention on mealtime behavior of people with dementia who wander	3 participants (2 women, 1 man) Inclusion criteria: medical diagnosis of Alzheimer Disease, with the habit of wander and table-leavers, consenting proxy, English-speaker, independently ambulatory and restraint-free, MMSE less than 24/30, Eating Behavior Scale score of 12/18, sight and hearing sufficient for everyday communication, and a recorded recent weight loss	Systematic reinforcement of sitting-at-table behavior by the resident using two communication strategies: focused conversation about the meal, eating and social comments related to the mealtime experience, and specific elements of social behavior (smiling, eye contact)—daily for 5 days in the first 20 minutes of the mealtime	Table-leaving (frequency and duration) Food consumption Body weight MMSE	All cases were able to sit at the table longer and eat more food during the intervention, while body weight for all cases remained stable throughout the study Two of the three cases left the table fewer times during the intervention There were no statistically significant changes in proportion of fluids consumed in any case
Cleary et al., 2012 (Canada)	Within group, repeated measures	To assess the effects of reminiscence on consumption of food by residents at-risk for nutritional decline and to examine the relative effects of conversation and cueing on their food consumption	7 residents (5 women, 2 men) Mean age: 86.1 years Inclusion criteria: diagnosis of dementia (moderately to severely impaired in cognitive function); able to sit upright to eat; spoke English fluently; institutionalized for at least 3 months; adequate vision and hearing for normal conversation; not taking medications aimed at appetite stimulation; physically able to self-feeding	Structured reminiscence conversation protocol to verbal cueing and prompts to eat Phase A (baseline): typical level of mealtime support, including feeding of residents Phase B: researchers carried out either a social reminiscence protocol at mealtimes (without staff support) Phase C: structured verbal cueing protocol at mealtimes (without staff support)	Amount of food eaten during each meal (weight)	There was no significant difference in intake as a function of either treatment condition as compared to baseline In the conversation/reminiscence condition, participants ate 5% more food on average than during the verbal cueing condition In the reminiscence condition, 5 of 7 participants ate more as compared to the cueing condition
Coyne & Hoskins, 1997 (USA)	Experimental—Pretest-posttest	To determine the short- and long-term efficacy of directed verbal prompts and positive reinforcement on the level of eating independence of residents	24 residents (all women) Age from 68 to 96 years Inclusion criteria: diagnosis of dementia, consume 3 meals in the communal dining room, eat at least half of their meals without staff assistance	Directed verbal prompts to each experimental group and positive reinforcement the eating tasks were completed 13-days, including a pretest, treatment and 2 posttests	Level of eating independence scale	Significant differences were found in eating performance but not in frequency Experimental groups retained treatment at both posttests
van Ort & Phillips, 1995 (USA)	Experimental	To test the efficacy of contextual and behavioral interventions design to promote functional feeding and maintain adequate nutritional status of a sample of residents	**7 residents** (5 women, 2 men) Age from 65 to 93 Inclusion criteria: required feeding assistance by a caregiver, able to sit in a chair for feeding, responsive to human interaction, not usually restrained during feeding, not usually combative **18 staff feeders:** registered nurses, licensed practical nurses, and nursing assistants	Contextual intervention: noise and distraction from all sources was minimized; food was placed directly in front of residents and arranged on a placemat; position functionally impaired residents next to self-feeding residents; avoid staff feeder interruptions during meal Behavioral intervention: using simple verbal or tactile prompts immediately by offering food; repeating instructions as cues; pantomiming desired behaviors; reinforcing eliciting behaviors by starting a feeding episode; reinforcing self-feeding attempts through praise and positive facial expressions; using verbal and tactile reinforcement; using sustaining behaviors to maintain the continuity of the meal	Feeding Trace-Line Technique Body weight MMSE	Both interventions resulted in feeding-related interpersonal contact between residents and feeders Both interventions resulted in a better match between the functional abilities of the resident and the level of assistance offered by the feeder Both interventions resulted in maintenance of the residents’ nutritional status as evidenced by no change weight

Abbreviations: BIMS = brief interview for mental status; EdFED = Edinburgh Feeding Evaluation in Dementia; MMSE = Mini-Mental State Examination.

#### Impact of mealtime assistance

Three of the 4 mealtime assistance interventions included only residents as participants of the studies, reporting the impact of interventions on them [49, 50, 63]. The other study included residents and "staff feeders" but demonstrated the intervention’s impact only on residents [64].

Studies that only included residents had the objective of improving food intake and some behaviors, such as wandering and the ability to self-feed. Food intake was improved in a contextual and behavioral intervention [64]. One study did not impact residents’ oral intake, which remained the same or showed no significant differences in pre-post intervention behavior [50]. A study that aimed to assess the residents’ level of eating independence evidenced the positive impact of directed verbal prompts and positive reinforcement in making residents improve their levels of independence to eat, in addition to maintaining nutritional status [63]. Although the authors integrated registered nurses, licensed practical nurses, and nursing assistants among the participants, the impact of the intervention on these professionals was not evidenced, only indicating the potential benefit for the staff in having residents with less eating dependence [64].

### Staff training

Five studies (15.1%) presented training programs developed for nursing homes staff to support residents’ mealtime support needs (Table 3). Feeding skills of staff were the focus of the training program in three studies. One study included 3 hours of in-service classes to teach a protocol for mealtime support and 1 hour of hands-on training, and its effectiveness was evaluated through measures of food acceptance by residents, and knowledge and attitudes of staff [55]. Two studies used technology as the method of delivering training to staff. One was a web-based educational intervention using a problem-solving approach associated with the use of hand feeding techniques [51]. Its effectiveness was measured through the staff’s skills and the food intake and feeding behaviors of the residents during meals. The other study developed a mobile application for staff education on meal attendance and its preliminary effects were identified in staff and in residents [17].

**Table 3:** Staff training interventions studies (n = 5).

Study	Design	Aim(s)	Population	Intervention	Outcome Measures	Main results
Batchelor-Murphy et al., 2015 (USA)	Feasibility study	To test a web-based version of a dementia feeding skills educational intervention, and to examine its efficacy	**10 residents** (5 in control group, 5 in intervention group) Inclusion criteria: 65+ years old, institutionalized for at least 6 months, medical diagnosis of dementia, had a legal proxy to sign informed consent, required some level of feeding assistance, dependent for ADL, MMSE score of 19/30 or lower **35 staff members** (34 women) Certified Nursing Assistant, Licensed Practical Nurse, Registered Nurse Age ranged 21 to 60 Inclusion criteria: work at morning shift, employee for the previous 30 days	30-min narrated PowerPoint presentation, followed by a 4-min video) on mealtime difficulties using the C3P model + three hand feeding techniques + in-person group coaching sessions during the lunch meal after training	EdFED Food intake record NH staff knowledge and self-efficacy of feeding assistance Time providing feeding assistance Feeding Skills Checklist	Aversive feeding behaviors increased in both groups of residents. The intervention staff increased the amount of time spent with meal assistance, and food intake doubled. In the control group, less time was spent providing assistance and meal intake decreased
Chang & Lin, 2005 (Taiwan)	Quasi-experimental	To provide a feeding skills training program for nursing assistants and to test its effects on the outcomes of staff and residents	**36 residents** with dementia and eating problems needing assistance Mean age 84.2 in treatment group and 72 years in control group **67 nursing assistants**: 31 in the treatment group—all women; 36 in the control group—2 men	3 hours of in-service classes (overview of dementia, etiology, and behaviors of feeding among dementia residents and protocol for feeding dementia residents) and 1 hour of hands-on training Written manual of this feeding skills training program was provided	Formal Caregivers’ Knowledge, Attitude and Behaviors toward Feeding Dementia Residents EdFED Total eating time Food intake	The treatment group had significantly more knowledge, more positive attitude and better behaviors than the control group after the intervention Residents in the treatment group had significantly longer total eating time and higher EdFED than the control group. There was no significant difference on food intake between the two groups
Jung et al., 2020 (Korea)	Mixed methods	To develop a mobile application for meal assistance training and to test the feasibility of its usage by direct care workers, as the preliminary effectiveness of this intervention on staff and residents	**23 residents** (82.6% women) Mean age: 86.09 years Inclusion criteria: diagnosis of dementia, living in the NH > 6 months **23 direct care workers** (all women) Mean age 60.83 years Inclusion criteria: work for more than 6 months on NH, possess an Android-based smart phone	Mobile application with 4 sessions: premeal assistance, midmeal assistance, post meal assistance, and feeding-related issues Nine minutes of direct education using PowerPoint, and instructions for using the APP Four weeks of intervention	Mucus Life machine Eating Behavior Scale Eating time Formal Caregivers’ Attitude and Behavior toward Feeding Dementia Residents Questionnaire Observation Checklist	Direct care workers reported that the most helpful educational content was “actual meal assistance” There were no significant differences between the APP pre- and post-intervention regarding residents eating behavior, oral moisture, or mealtime length There was no significant difference in staff’s attitudes or knowledge
Lin et al., 2010 (Taiwan)	RCT	To investigate the effectiveness of training of spaced retrieval (SR) and Montessori-based activities in decreasing feeding difficulty and nutritional status for residents	**85 residents** (45 women, 40 men) Mean age: 81.18 years Inclusion criteria: diagnosed with dementia, scored ≥ 2 on the EdFED, able to stay in the institutions during the entire study period; MMSE 10–23 3 groups: spaced retrieval, Montessori and control	35–40 min sessions, 3 times per week, for 8 weeks Spaced retrieval group: training in eating procedure and eating behavior Montessori group: hand-eye coordination, scooping, pouring, and squeezing activities Control group: daily routine normally followed by the institution	MMSE Barthel index EdFED MNA BMI Meal duration and amount consumed	EdFED scores and assisting feeding scores for the SR and Montessori-based activity groups after intervention were significantly lower than that of the control group Frequencies of physical assistance and verbal assistance for the Montessori-based activity group after intervention were significantly higher than that of the control group MNA in the SR group was significantly higher than that of the control group, while MNA in the Montessori-based activity group was significantly lower than that of the control group
Wu et al., 2018 (Canada)	Mixed methods	To determine if the mealtime experience could be modified with the CHOICE Program, and how program components needed to be adapted and/ or if new components were required	**64 residents** (70% women) Mean age: 85 years **16 team members:** 10 Personal Support Workers, 3 Dietary Aids, 2 Registered Practical Nurse, 1 Recreational Therapist **5 members of home management:** 2 home area coordinators, 1 Director of Food Services, 1 Assistant Director of Food Services, 1 Director of Care, 1 quality indicators manager	Education session and training modules: 45 min Staff Huddles and Huddle Diary: 5–10 min./huddle; 1x week or as needed Visual Reminders: 1 poster/week; 2–3 posters per dining room or as needed Continuous Feedback: Comprehensive report based on Mealtime Scan Data CHOICE Coach: in-person visit: 5–7 h per home area	Mealtime Scan Cognitive Performance Score Activities of Daily Life—Long Form Semi-structured interviews Brief qualitative comments	Physical and overall mealtime environment ratings showed improvement over time Interviews revealed in-depth insights: i) Knowing the context and culture to meet staff and resident needs; ii) Getting everyone on board, including management; iii) Keeping communication lines open throughout the process; iv) Sharing responsibility and accountability for mealtime goals and challenges; v) Empowering and supporting staff’s creative mealtime initiatives

Abbreviations: ADL = activities of daily living; MMSE = Mini-Mental State Examination; C3P = change the person, change the people, or change the place; EdFED = Edinburgh Feeding Evaluation in Dementia; NH = nursing home; APP = application; RCT = Randomized controlled trial; MNA = Mini-nutritional assessment; BMI = Body mass index

One study was a staff training based on spaced retrieval and Montessori activities and it was evaluated how these activities affected the mealtime support needs and nutritional status of residents [52]. The last study evaluated how a previously established program, based on relationship-centred care, could change the residents’ dining experience [65]. This study is the only one analyzed in this review that includes results for the residents, care staff, and nursing home administrators.

#### Impact of staff training

Studies with staff training were more comprehensive in relation to participants, involving residents, care staff, and nursing homes administrators. Three studies aimed to evaluate the effectiveness of feeding skills training. The impact on residents was demonstrated through food intake, while in the staff aspects such as knowledge, self-efficacy, attitude, and behavior towards residents’ mealtime support needs were evaluated. The impact on staff was evidenced in 2 studies, noting more knowledge, more positive attitude, and better behaviors, and in one of them, there was also an improvement in the residents’ food intake [51]. In one of the studies, despite the positive impact on staff, food intake, and eating behaviors of residents did not change [55]. The intervention through a mobile application did not present significant results either for the residents or for the staff [17].

One study considered only the impact of the intervention on the mealtime support needs and nutritional status of residents, with satisfactory results, where residents presented a reduction in mealtime support needs and maintenance of nutritional status [56].

Only one intervention of all the studies analyzed in this scoping review, considered 3 groups that are directly or indirectly involved at mealtimes: residents, care staff, and nursing homes administrators. The study aimed to assess whether a program had an impact on the dining experience, and staff impressions were collected through semi-structured interviews and qualitative comments [65].

### Multicomponent interventions

The remaining six studies (18.3%) reported interventions that combined more than one category within the scope (Table 4). One study combined environment modifications with a workshop aimed at altering “eating” into meaningful dining experiences and looked at the impact on residents’ food intake and behavior, and staff’s care performance [54]. One study evaluated the effect of “family-style” meals with serving dishes instead of prepared plates combined with in-service staff training on prompting and praising appropriate resident behavior [66].

**Table 4:** Multicomponent interventions studies (n = 6).

Study	Design	Aim(s)	Population	Intervention	Outcome Measures	Main results
Altus et al., 2002 (USA)	Experimental	To examine the impact of using serving dishes versus prepared plates on participation in mealtime tasks by residents	**5 residents** (all women) Mean age: 80 years Inclusion criteria: diagnosis of dementia, MMSE mean score 8, ambulatory, non-required skilled nursing care **1 certified nursing assistant**: 24 years old	Prepared Plates (baseline condition)—each resident’s plate was prepared in advance of the meal Family-style Meals: food was presented in communal serving dishes instead of preparing individual resident plates Staff Training: 45 min in-service training session on prompting and praising appropriate resident behavior	Checklist of tasks Resident communication (appropriate or inappropriate) Number of praise statements made by the staff during the lunchtime observation Staff satisfaction with residents’ levels of participation and communication, and overall satisfaction with lunchtime	Baseline: very low rates of appropriate communication (5.5% of intervals) and mealtime participation (10% of tasks) Family-style meal: participation doubled (24%) and communication (10.6%) but were still low Family-style Meals + Staff Training: participation increased to 65% of tasks and appropriate communication increased to 18% of observations
Cartwright et al., 2022 (Australia)	Observational	To assess a Montessori mealtime intervention impact on person-centred care for dementia residents	17 residents (no information on gender and age) Inclusion criteria: living with memory loss, with dementia symptoms ranging from mild to severe on MMSE **10 regular care staff** (no information on gender and age)	Staff-education in a Montessori-based model of care–*Care with Purpose*–with focus on: environmental modifications, policies and procedures, training and communications processes	Video-coding protocol with 4 categories: providing choice and preferences, promoting the social side of eating, supporting independence, and showing respect towards the residents	Significant positive changes in staff-resident interactions, choice behaviors, and support for mealtime independence. These improvements were observed consistently over time, indicating the sustained effectiveness of the intervention. The findings also highlighted the complexity of mealtime care and emphasized the importance of fostering a culture change in this context.
Lin et al., 2011 (Taiwan)	Experimental crossover	To investigate the efficacy of a Montessori intervention on improving eating ability and nutritional status of residents	29 residents (12 women, 17 men) Mean age: 82.9 years Inclusion criteria: diagnosed with dementia, scored ≥ 2 on EdFED, MMSE ranging from 10–23	• 30 minutes daily sessions, 3 days/week, for 8 weeks • 24 activities of procedural movements (hand–eye coordination, scooping, pouring, squeezing and matching) • Sensory stimulation with music • Review of the day’s activity	EdFED Eating Behavior Scale Mini-nutritional assessment Body mass index Stopwatch (meal duration)	Significant reduction in the EdFED score for the Montessori intervention period but not for the routine activities period. The mean differences for the EBS score, self-feeding frequency and self-feeding time were significantly higher than those of the routine activities period. Except for the MNA score post-test, no significant differences for any other variables were found for the routine activities period.
Perivolaris et al., 2006 (Canada)	Pre- and postintervention with repeated measures	To describe an Enhanced Dining Program and to discuss its effectiveness	**11 residents** (3 women, 8 men) Mean age: 84.6 years Inclusion criteria: MMSE average score 13.9, diagnosed with dementia, resident for at least 1 month, physically able to self-feed and spoke or understood English **7 staff members** (all women) Registered nurses and practical nurses, activity aid, recreation therapy assistant, employed for at least 3 months	**Enhanced dining program:** a pleasant physical environment of dining room + staff providing verbal cueing and prompting throughout the meal. Once a week, for 4 months **Workshop:** “E”-Dining Education Program. E = environment, enablement, engagement, eating, evaluation, and education + a review of best practices that promote caring, choice, and independence during the dining experience. 1 day.	Food and fluid intake Feeding Abilities Assessment Pittsburgh Agitation Scale Checklist of enabling behaviors	Positive impact of the Enhanced Dining Program on resident caloric intake. The residents’ improved functioning both from a physical and a social standpoint contributed to the greater staff satisfaction. The combination of environmental modifications and staff education produced greater results than changes to the physical dining space.
Rehman et al., 2023 (UK)	Single-case experimental ABA design	To test a spaced retrieval intervention for the alleviation of mealtime difficulties	8 residents (6 women and 2 men); Mean age: 78.5 years Inclusion criteria: diagnosis of dementia, ability to communicate effectively, pass one item spaced of retrieval screening and a reading test	40–60 minutes sessions on Mondays, Wednesdays, and Fridays for 8 weeks. Spaced retrieval activities related to recognizing mealtime, feeding themselves, eating and swallowing.	EdFED MNA Body mass index Realist evaluation Economic evaluation	Spaced retrieval showed a positive effect, and the effect sizes were medium. EdFED mean score between phase A1 and B was reduced. There was improvement in the BMI and MNA in all residents. Limited effectiveness of realist evaluation in identifying intervention success factors. Intervention cost per kcal: £47.62
Wu et al., 2014 (Taiwan)	Single-blinded, quasi-experimental with repeated measures	To examine the long-term effects of a standardized and individualized training sessions of spaced retrieval combined with Montessori-based activities on improving eating difficulties, eating amount and body weight of residents	90 residents (all men) Mean age: 82.9 years Inclusion criteria: diagnosed with dementia, EdFED ≥ 2, MMSE 6–23, passing a spaced retrieval screening test, able to speak Chinese.	Spaced retrieval combined with Montessori-based activities. 3 groups: standardized, individualized and control. 24 intervention sessions over 8 weeks	EdFED Eating amount Body weight Mini Mental State Examination	Participants who received the standardized/individualized interventions exhibited a significantly greater decrease in the frequency of eating difficulty across time than did the participants in the control group. The body weight of the standardized and individualized groups also increased significantly by 0.99 and 0.72, respectively, per time interval compared with that of the control group.

Abbreviations: MMSE = Mini-Mental State Examination; EdFED = Edinburgh Feeding Evaluation in Dementia; EBS = Eating Behavior Scale MNA = Mini-nutritional assessment.

One study was a Montessori intervention that involved sensory stimulation through music, procedural movements (hand–eye coordination, scooping, pouring, squeezing, and matching), and reviewing the day’s activities with the aim of improving the eating ability and nutritional status of residents [60]. A second multicomponent study also used Montessori-based activities, but combined with spaced retrieval activities, targeting improving mealtime support needs, food intake, and body weight of residents [53]. Other study assessed the impact of a Montessori mealtime intervention on person-centered care by associating environmental modifications, procedural changes to the mealtime services and implementation of policies according to Montessori principles, to staff training and communications methods [67].

Lastly, a spaced retrieval intervention was tested, through sections with activities created to help residents re-learn a fixed series of actions related to recognizing mealtime, feeding themselves, eating and swallowing, while controlling environment settings (lighting, noise) [68].

The multicomponent interventions were equally divided regarding the inclusion of residents and staff as participants. Three studies that included only residents had similar objectives, which included improving oral intake and eating abilities [53, 60], relieving the mealtime support needs [68]. The impact of the interventions was positive, as there was an increase in oral intake, body weight, and a reduction in inappropriate mealtime behaviors, with a consequent improvement in the ability to eat. In these three interventions, Montessori-based and spaced retrieval activities were used.

#### Impact of multicomponent interventions

Interventions that included residents and staff among participants had different objectives and both associated changes in the environment with staff training. The study that aimed to increase residents’ participation in mealtime tasks resulted in greater involvement and more adequate communication between residents, in addition to the care staff being more satisfied with the higher level of residents’ participation during mealtimes [66]. Another study aimed at reducing agitation and improving eating abilities and oral intake of residents also showed a positive impact, with evidence of increased food and fluid intake and better physical and social functioning. Regarding the staff, there was a report of increased satisfaction with the use of ability-enhancing interventions and best practices [54]. The study that evaluated a Montessori mealtime intervention in person-centered care, showed a significant positive change in staff-resident interactions, opportunity for residents’ choice, and support for mealtime independence [67].

## Discussion

This scoping review analyzed the literature on interventions for mealtime support needs of people with dementia, and its impacts on residents, care staff, and care context/environment. The social ecological model was the framework used to connect the perspectives of these three stakeholders’ groups since it is helpful to understand the interaction of factors influencing challenges during mealtime for residents. Overall, several studies have described interventions designed to address residents’ mealtime support needs, leading to better food intake and nutritional status, but also to make mealtimes a more pleasant time, adjusting the residents’ behavior.

Thirty-three mealtime interventions were identified in this review and were classified into four types according to the nature of the strategies chosen by the authors. Most of the interventions were **environmental**, with changes in the dining room based on use of music, meal service delivery and presentation of food, placement of aquariums, and adequacy of lighting and noise. Some interventions focused on improving the quality of **mealtime assistance**, while others consisted of **staff training** with different techniques. A fourth group of interventions can be considered **multicomponent** as they include more than one type of strategy.

Overall, the objectives of the selected studies were to test the effectiveness of different types of intervention in two main dimensions of the mealtime support needs: food intake and challenging behaviors of the residents, specifically agitation. Although the common objective of the studies was to test the effectiveness of the interventions, they differ between the proposed intervention actions and in relation to the outcome measures selected to identify improvements in the participants involved. The absence of a consensual definition of the concepts of eating/feeding/mealtime difficulties/support needs may be one of the reasons for interventions focusing on different aspects, be it the person with dementia, the staff, or the environment.

Based on the definitions presented in the introduction of this article, the studies analyzed in this review predominantly used the concept of eating (n = 22). For the remaining articles six used the concept of mealtimes [57, 59, 62, 66–68]; and 5 studies used the concept of feeding [17, 46, 51, 55, 64]. It was expected to find a relationship between the studies that used the concepts of feeding or mealtime support needs and outcomes for both residents and staff, but it was not possible to establish such association. Most studies (n = 23) demonstrated the effectiveness of interventions through outcomes presented by residents. As expected, 19 of them referred to the term eating difficulty [38–45, 47, 49, 50, 52, 53, 56, 58, 60, 61, 63, 65]. Despite presenting only residents’ outcomes, one study [46] considered the context as feeding support needs and two others as mealtime support needs [57, 59]. One study [68] included an economic evaluation of intervention costs related to daily calorie and protein intake.

Nine studies assessed the effectiveness of the proposed intervention presenting outcomes for residents and staff. Four of these studies [17, 51, 55, 64] were based on the concept of feeding support needs, two [37, 54] were about eating support needs, and three [62, 66, 67] were about the context of mealtime support needs. Only one study showed the impact of the intervention on residents, nursing staff and also reported how nursing home administrators comprehend this impact [65].

Since malnutrition and weight loss are prevalent in residents, and associated with functional decline, increased hospitalizations, and more dependence on activities of daily living [69], it is to be expected that interventions will seek results in improving food intake. Food and liquid intake have been recognized as an important interventional target for improving nutritional status of residents, being a concern for the development of interventions [70].

Cognitive impairments typically found in residents cause difficulties in performing tasks such as self-feeding, handling cutlery, or behaving at the table. They also induce to depression, aggressive behavior, agitation, apathy, wandering, and emotional distress that can cause or intensify mealtime support needs [71, 72]. Interventions that propose to create a calm environment for mealtime, leading to better residents’ behaviors, need to consider that the eating behaviors of residents are likely motivated by personal antecedents and environmental factors and must be considered as [73].

This scoping review identified several ways to intervene in the mealtime support needs of residents, either by directly support resident independent eating or improving staff’s ability to provide mealtime assistance or increase food intake. The use of the concept of mealtime support needs was not consensual among the results, and studies that referred to feeding support needs or eating support needs were also analyzed. The interventions mainly focused on dining room changes/adaptations, mealtime assistance methods, training of nursing staff, or even a combination of both.

As mealtime involves factors related to residents, staff, and the environment where the meal occurs, including institutional factors such as organizational culture, limiting the scope of interventions can lead to the improvement of isolated factors, like better food and liquid intake. From the perspective of the Social Ecological Model, mealtime support needs can be interpreted and managed through multiple factors that influence mealtimes. Besides intrapersonal factors and the interaction between residents and staff, policies, routines, and institutional culture, at a macro level, must be considered in an intervention [74]. Most of the interventions mapped in this scoping review centered their objectives at the intrapersonal, interpersonal, and environmental levels (care context), focusing essentially on residents and care staff separately, and also on the dyad formed between these two. These studies demonstrated the effectiveness of interventions in outcomes directly related to the residents, and a few to the staff. Interventions focused only on isolated factors may not be completely effective when considering the concepts used, whether eating, feeding, or mealtime support needs.

Only one study [65] analyzed the impact of the intervention at the macro-level (institutional policy/systems factors) and indicated the importance of the nursing home administrator’s integration in the intervention. This integration can result in the development of better strategies to support the care team for continuity of the intervention. Multicomponent interventions allow for a comprehensive and multidimensional approach to the challenge. Rather than focusing only on resident factors, a broader perspective that considers interactions between residents, care staff, and nursing home administrators in the care environment is encouraged. By addressing challenges and solutions at multiple levels simultaneously, interventions can be more comprehensive, promoting a better mealtime experience for residents and potentially improving their nutritional status and general well-being. Including, for example, staff training or even assessing the involvement of the administrators in these issues, the outcomes could be much more comprehensive.

There is a lack of information about the role of nursing home administrators in handling mealtime support needs. Future research should explore the role of nursing home administrators in managing residents’ mealtime support needs, analyzing institutional policies, care protocols, and standards of good practice in residential care settings. Administrators play a key role in decision-making processes and shape the institutional culture, and their support is essential for the successful integration of interventions. By understanding administrators’ viewpoints, researchers can tailor interventions that align with the existing culture, potentially increasing adhesion from staff and facilitating smoother implementation. Therefore, the results of this review may inform future research and also in the creation of care protocols within institutions to minimize mealtime support needs for people with dementia.

### Limitations

Even though this scoping review was conducted with a rigorous and methodical process there are still some limitations that need to be acknowledged. Only five databases were used in this scoping review, and despite the search being carried out in four languages (English, French, Spanish, and Portuguese) some studies might have been missed. Even with all efforts, some articles published in non-open access journals may not have been reached in this study. Another limitation of this study may be the non-regular use of the terms eating, feeding or mealtime difficulties/support needs across the studies. The variety of strategies found, as well as the diversity of outcome measures applied to each study, limited a fairer comparison between interventions, as well as making it difficult to identify the most effective intervention.

## Conclusion

This article broadened the comprehension of the effects of mealtime interventions as perceived by crucial stakeholders: residents, care staff, and nursing home administrators. Of the studies identified and analyzed, the interventions were grouped into four categories: environmental, mealtime assistance, staff training, and multicomponent. Most interventions analyzed the impact only in residents, namely in oral intake and behavior of people with dementia, specifically agitation. Unlike many previous reviews that predominantly focus on residents, this scoping review emphasizes the impact of interventions on care staff, revealing correlations between staff training and improved knowledge and attitudes.

This review underscores the necessity for mealtime intervention studies to assess outcomes from a comprehensive or multi-level perspective. Studies that assess the impact on administrators are necessary to understand the perspective of different hierarchical levels of an organization on mealtime support needs. The findings of this scoping review can support the creation of new supportive programs, or strategies to improve mealtime experience with positive impacts according to the reality and needs of each person or institution.

## Supporting information

S1 Checklist PRISMA 2020 checklist. (PDF)
